# Structural Basis for Elastic Mechanical Properties of the DNA Double Helix

**DOI:** 10.1371/journal.pone.0153228

**Published:** 2016-04-07

**Authors:** Young-Joo Kim, Do-Nyun Kim

**Affiliations:** 1 Department of Mechanical and Aerospace Engineering, Seoul National University, Gwanak-ro 1, Gwanak-gu, Seoul, 08826, Republic of Korea; 2 Institute of Advanced Machines and Design, Seoul National University, Gwanak-ro 1, Gwanak-gu, Seoul, 08826, Republic of Korea; Institut Pasteur, FRANCE

## Abstract

In this article, we investigate the principal structural features of the DNA double helix and their effects on its elastic mechanical properties. We develop, in the pursuit of this purpose, a helical continuum model consisting of a soft helical core and two stiff ribbons wrapping around it. The proposed model can reproduce the negative twist-stretch coupling of the helix successfully as well as its global stretching, bending, and torsional rigidities measured experimentally. Our parametric study of the model using the finite element method further reveals that the stiffness of phosphate backbones is a crucial factor for the counterintuitive overwinding behavior of the duplex and its extraordinarily high torsional rigidity, the major-minor grooves augment the twist-stretch coupling, and the change of the helicity might be responsible for the transition from a negative to a positive twist-stretching coupling when a tensile force is applied to the duplex.

## Introduction

Recent advances in single-molecule experiments have thrown new light on the mechanics of the DNA double helix through direct manipulation of individual DNA molecules and characterization of their structural properties [1–3]. In particular, the elastic response of DNA double strands has been extensively studied, revealing their unique mechanical properties including the extraordinarily high torsional rigidity (approximately twice the bending rigidity) [4] and the counterintuitive overwinding behavior under tension [4–6]. Numerous experiments have also demonstrated that these elastic properties are closely related to the helical conformation such as the axial rise (the distance between neighboring base-pairs along the helical axis) and the helical repeat (the number of base-pairs per one helical turn) that may vary with, for example, specific base sequences [7], dinucleotide steps [8], neutral or charged modification of base-pairs [9], and binding of small molecules [10, 11]. However, the structural origin of these intriguing duplex properties remains elusive.

In this article, we study the principal structural features of the duplex and their plausible role on its elastic mechanical properties using a helical continuum model where DNA double helices are treated as elastic helical solids with a polygonal cross-section. It is noteworthy that a simple isotropic cylinder model cannot reproduce the exceptionally high torsional rigidity and negative twist-stretch coupling [4]. Also, unlike the elastic rod model [12–17], arguably the most popular modeling approach to DNA mechanics and widely used to study highly nonlinear behaviors of the DNA duplex for given stiffness coefficients of stretching, bending and twisting, our helical model seeks to identify the primary structural features governing those stiffness values and understand how they are determined better. We perform a comprehensive computational analysis using this model constructed by varying the helicity, the stiffness of phosphate backbones, and the major-minor groove pattern systematically.

## Methods

### Helical continuum model for the DNA double helix

**Fig 1:**
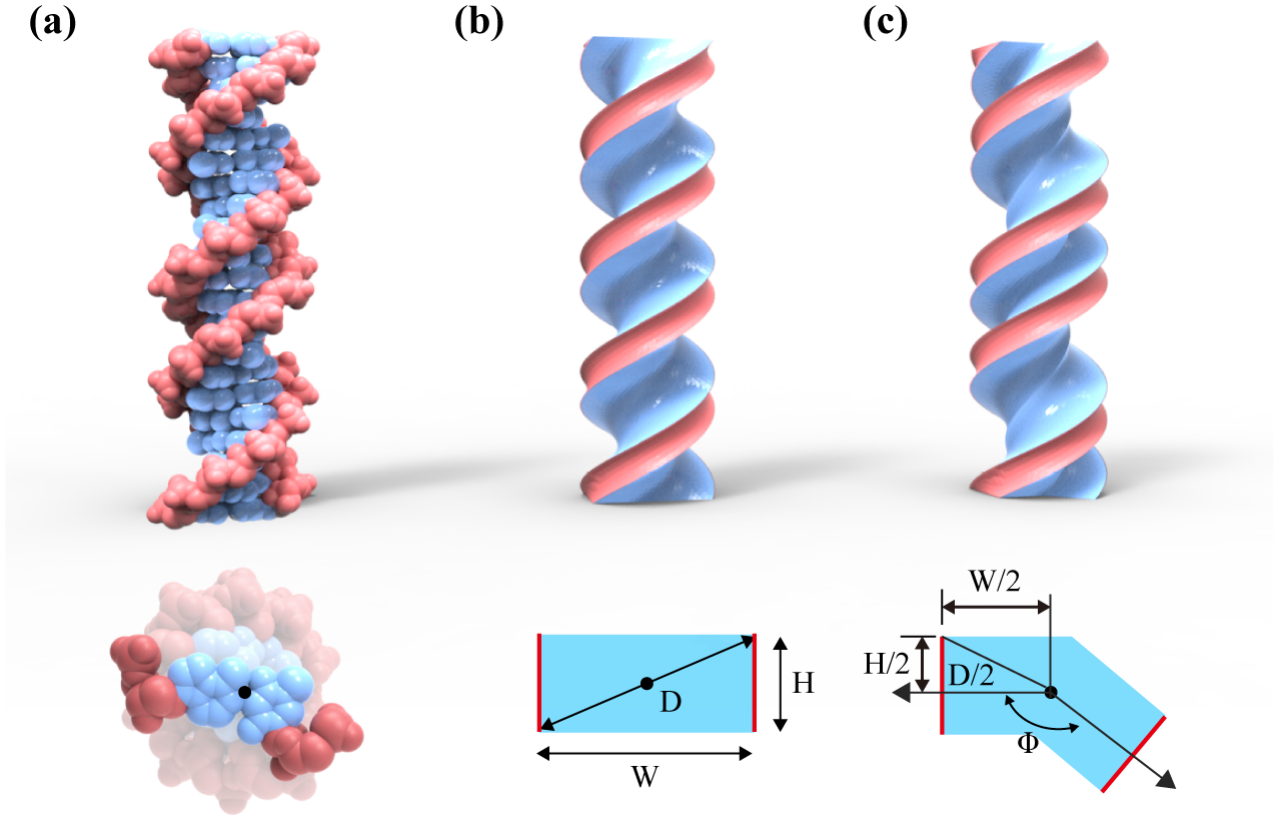
Helical continuum model. (a) The atomic structure of the B-form DNA double helix, (b-c) helical continuum models consisting of the soft core (blue) and two stiff ribbons (red) without and with major-minor grooves. The cross-section of the helix is parameterized using either the width (W) and the height (H) or the diagonal length (D) and the aspect ratio (AR = W/H) with the groove angle (Ф).

The helical continuum model consists of a soft core and two thin, stiff ribbons wrapping around the core (Fig 1). The core structure is generated via helical sweep of a cross-section along the helical axis with a constant pitch assuming the straight mean conformation by neglecting any intrinsic, sequence-dependent curvatures in reality [13, 18, 19]. The core has a simple rectangular cross-section parameterized using the diagonal length (D) corresponding to the helix diameter and the aspect ratio (AR) that is the ratio of the width (W) to the height (H) (Fig 1b). Major-minor grooves are included in the model by using the groove angle (Ф) resulting in a kinked rectangular cross-section while keeping the cross-sectional area invariant to the groove angle (Fig 1c). Helical geometry of the model is described using the axial rise (ΔZ) and the twist rate (Δθ) that is the right-handed twist angle between neighboring base pairs. These parameters for the B-form DNA are used as the default values in our analysis that are Δθ = 34.29°/bp [20, 21] and ΔZ = 0.34 nm/bp [22, 23]. Two helical ribbons representing stiff phosphate backbones follow the helical path formed by two narrower edges of the core cross-section.

### Finite element model

Finite element (FE) models are constructed for various helical continuum models as follows. The core is assumed as a nearly incompressible elastic material described by its Young’s modulus (E_c_) as a free parameter with Poisson’s ratio (ν_c_) of 0.5 [24]. Helical ribbons are assumed to provide the stretching rigidity (S_r_) only. The core structure is meshed using ten-node tetrahedral solid elements while the ribbons are discretized using six-node triangular shell elements. The element size is determined based on convergence analysis to ensure sufficiently high solution accuracy with reasonable computational cost in our exhaustive parametric study (Fig A in S1 File). The parameters used to construct FE models are summarized in Table A in S1 File.

### Calculating the elastic mechanical properties

Finite element analysis, which has been useful for investigating the mechanics of the DNA double helix [25, 26], is performed to calculate the linear elastic mechanical properties of various helical continuum models constructed with systematically varied geometric (D, AR, Ф, Δθ, and ΔZ) and material (E_c_ and S_r_) parameters using the commercial finite element analysis software ADINA (ADINA R&D Inc., Watertown, MA, USA). We investigate the effect of these model parameters particularly on four representative duplex properties: the stretching rigidity (S) [27–29], the bending rigidity (B) [28–32], the torsional rigidity (C) [31, 33–36] and the axial displacement coupled to twist (ΔL_c_). The stretching rigidity is calculated using S = F_a_ L where L is the helix length and F_a_ is the reaction force when a unit axial displacement is applied at one end of the helix while fixing the other end. Similarly, the bending rigidity and the torsional rigidity are computed using B = M_b_L and C = M_t_L, respectively, where M_b_ is the reaction moment resulted from a unit bend angle applied and M_t_ is the one when a unit twist angle is applied. ΔL_c_ is the axial displacement when a unit twist angle is applied to the helix, which provides the twist-stretch coupling constant (g) [4–6] through g = –SΔL_c_ [4]. We use 210-bp-long helical models to avoid any length dependence in mechanical calculations (Fig B in S1 File) that are longer than the persistence length of the B-form DNA [32].

## Results and Discussion

### Effect of the helicity

**Fig 2:**
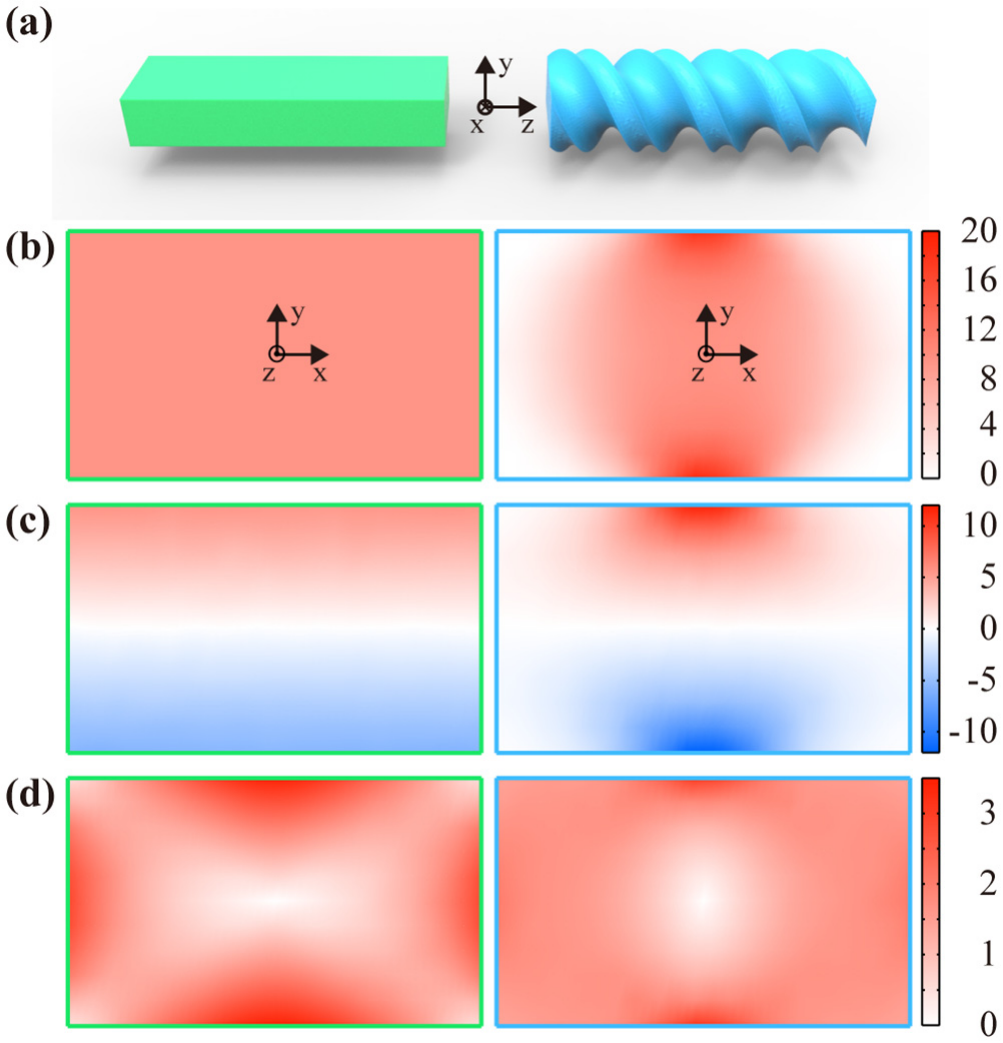
Stress distributions on the cross-section of the rectangular prism (left) and of the bare helix model without backbone stiffness (right). (a) Three-dimensional structures, (b) the axial stresses under tension, (c) the axial stresses under bending, and (d) the in-plane shear stresses under torsion. Results are calculated using E_c_ = 668 MPa, D = 2.4 nm, and 1/AR = 0.6 with the default helical parameters for the helical continuum model. Stresses are given in MPa.

We first investigate the bare helix consisting of the core only without the stiff ribbons. The helical model with the helicity of the B-form DNA exhibits lower rigidities in stretching and bending (36% in S and 21% in B, for instance, when D = 2.4 nm and 1/AR = 0.6) than those of the rectangular prism structure while the torsional rigidity, which also has the dependency on the helicity (Fig C in S1 File), remains in a similar value at this specific helicity (Fig 2). Axial stresses in stretching and bending become distributed on a disk, formed by overlapping the cross-sections at various helical orientations, serving as an effective load-bearing core (Fig 2b and 2c). In-plane shear stresses under torsion, on the other hand, are concentrated on the edges due to the cross-sectional warping, but the level of concentration decreases with the helicity (Fig 2d). These intriguing features of helical structures render the importance of considering the effect of the helicity properly in the model.

**Fig 3:**
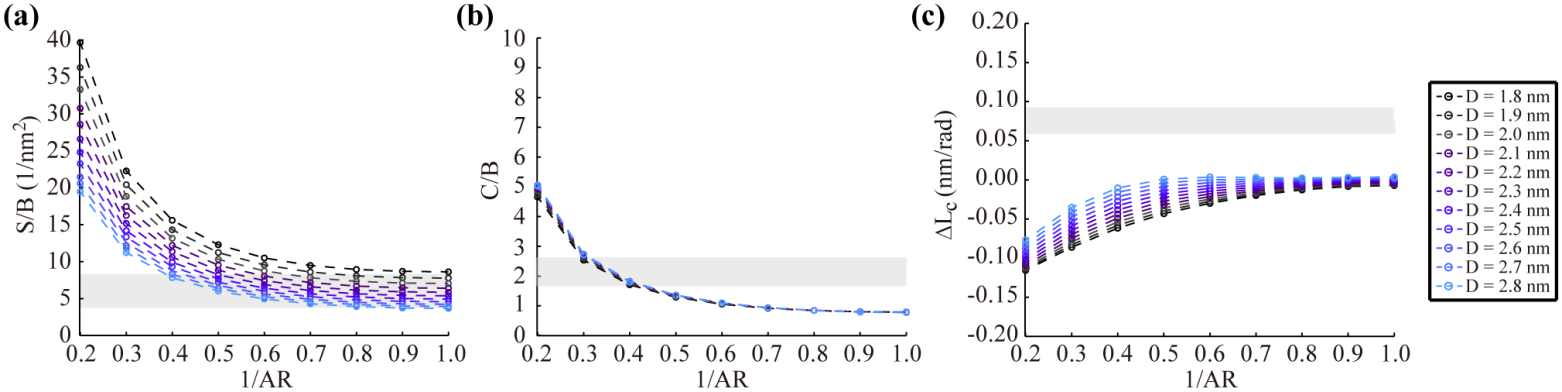
Mechanical properties of the bare helix model without backbone stiffness. (a) S/B depends on both D and AR while (b) C/B is insensitive to D. Shaded regions represent the range of experimental S/B and C/B values. (c) The bare helix model is not able to reproduce the positive, experimental ΔL_c_ values corresponding to the shaded region for the entire range of D and AR tested. Results are calculated using E_c_ = 668 MPa with the default helical parameters.

Structural rigidities of the bare helix depend certainly on the geometry of its cross-section. We construct various helical models using a range of D (1.8 ~ 2.8 nm) and AR (1.0 ~ 5.0) and examine the effect of these cross-sectional parameters on S/B and C/B of the helix that are independent of the core modulus E_c_. Both S/B and C/B show a similar dependency on AR, however, C/B is almost independent of D while S/B decreases with D (Fig 3a and 3b) that can be inferred from the rigidities of rectangular prism structures whose S/B and C/B are proportional to (1+AR^2^)/D^2^ and 1+AR^2^, respectively. Results demonstrate the limitation of the bare helix model clearly as it fails to predict the experimental S/B and C/B simultaneously using a single set of D and AR. A high AR is required to obtain the experimental C/B while a low one is necessary for the experimental S/B (Fig 3a and 3b). More importantly, the bare helix model is incapable of capturing the counterintuitive overwinding behavior of the real DNA duplex under tension as it predicts negative ΔL_c_ values for most combinations of D and AR tested for the model (Fig 3c).

### Effect of the phosphate backbone stiffness

**Fig 4:**
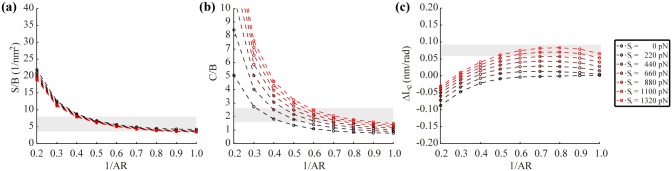
Mechanical properties of the helical model with backbone stiffness. (a) S/B is insensitive to S_r_ while (b) C/B depends on both S_r_ and AR. Shaded regions represent the range of experimental S/B and C/B values. (c) The helical model can reproduce the positive, experimental ΔL_c_ values corresponding to the shaded region for a wide range of S_r_ and AR values. Results are calculated using E_c_ = 668 MPa and D = 2.6 nm with the default helical parameters.

To overcome these limitations of the bare helix model, the stiff ribbons are added around the helical core. The stretching rigidity of the ribbons, S_r_, controls their extensibility when the duplex deforms and affects the overall mechanical properties considerably. Our parametric analysis shows that C/B increases with S_r_ significantly while S/B is almost insensitive to it (Fig 4a and 4b). This is mainly because the axial stiffness at the narrower edges of the cross-section barely affects S and B due to the helicity (Fig 2b and 2c). But, C varies with S_r_ as the torsion of the helix requires the length change of the ribbons that is restricted by their stiffness S_r_. Due to this structural effect of S_r_ on the overall duplex rigidities, both S/B and C/B within the range of experimental values can now be obtained simultaneously using a single set of D and AR. Moreover, the helical model can reproduce positive ΔL_c_ (or negative g) values consistent with the experimental observation as well in a broad area of the parametric space (Fig 4c and Fig D in S1 File), which is impossible using the bare model.

### Effect of the major-minor grooves

**Fig 5:**
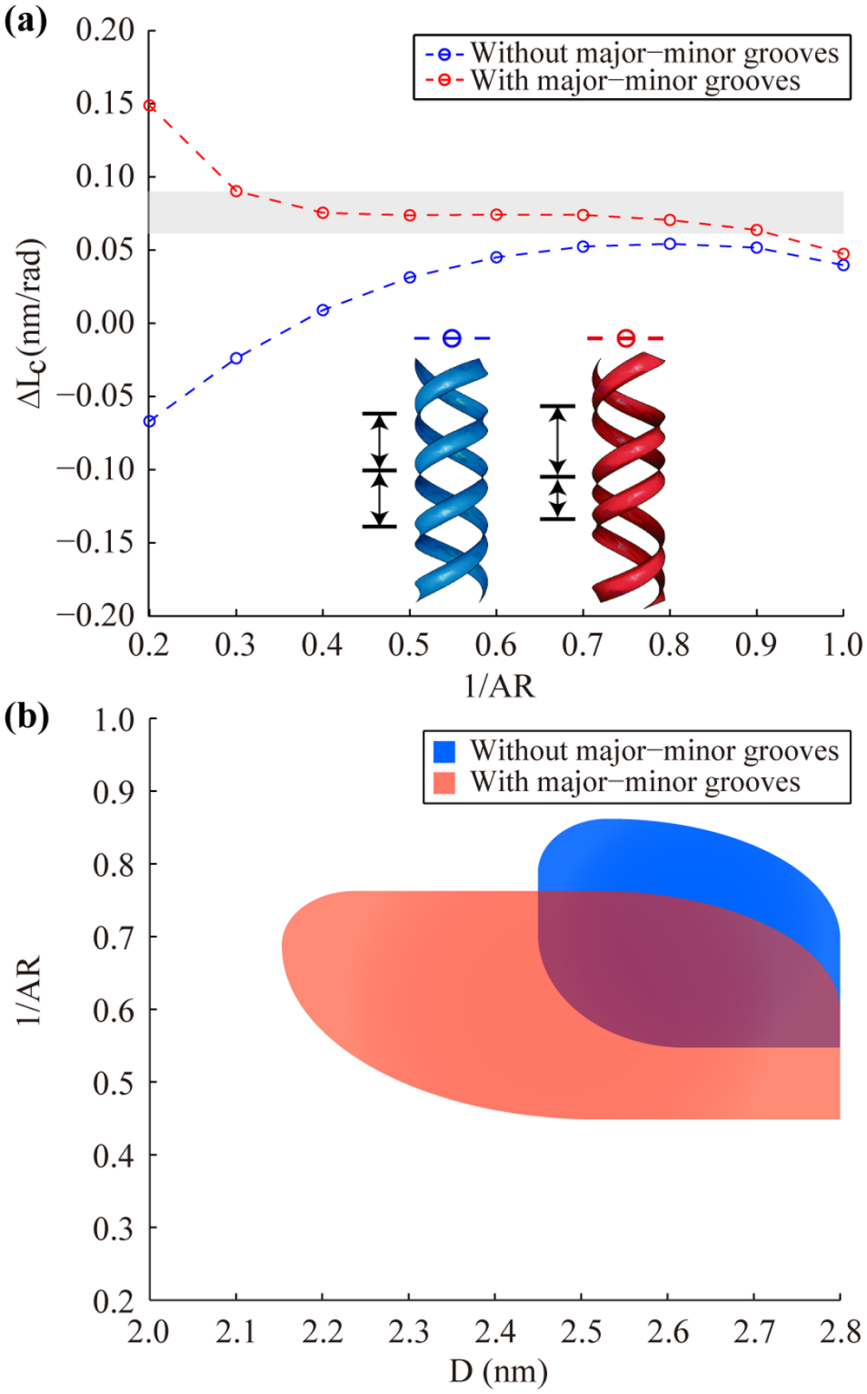
Effect of major-minor grooves. (a) The major-minor grooves increase ΔL_c_ for the entire range of AR. Shaded region represents the range of experimental values. Inset shows the ribbon configuration of the helical model without major-minor grooves (blue) and with major-minor grooves (red). Results are calculated using E_c_ = 668 MPa, D = 2.4 nm, and S_r_ = 1100 pN with the default helical parameters. (b) Feasible parameter values of D and AR necessary to reproduce the experimental mechanical properties of the B-form DNA when we use the helical model with major-minor grooves (red) and without them (blue).

Nevertheless, we have to choose D slightly larger than the diameter of the B-form DNA (1.85 ~ 2.40 nm depending on environmental conditions [37–39]) in this model to reproduce the target mechanical properties (S/B, C/B, and ΔL_c_) within the range of experimental values (Table 1), particularly due to the dependence of ΔL_c_ on D (Fig 5b). We found that this is largely because the model does not take the effect of major-minor grooves into consideration. Grooves are not evenly spaced in the DNA double helix because two phosphate backbones are not symmetrically positioned with respect to each other forming an angle of approximately 130° in the B-form DNA [40]. Their biological role has been studied intensively [10], but their structural role remains elusive.

**Table 1:** The mechanical properties and the diameter of the B-form DNA. For the isotropic cylinder model, we report C and g in consequence of choosing Young’s modulus and the diameter to satisfy S = 1100 pN and B = 230 pNnm^2^. For the helical model without major-minor grooves, the mechanical properties are calculated using D = 2.8 nm, 1/AR = 0.6, E_c_ = 411 MPa and S_r_ = 880 pN with the default helical parameters. For the helical model with major-minor grooves, the mechanical properties are obtained using D = 2.4 nm, 1/AR = 0.6, E_c_ = 668 MPa and S_r_ = 1100 pN with the default helical parameters.

	S (pN)	B (pNnm^2^)	C (pNnm^2^)	g (pNnm)	D (nm)
Experiments	900 ~1400 [27–29]	180 ~ 230 [28–32]	400 ~ 480 [31,33–36]	-100.0 ~ -70.0 [4–6]	1.85 ~ 2.40 [37–39]
Isotropic cylinder model	1100	230	153	0.0	2.26
Helical model without major-minor grooves	903	200	470	-85.6	2.80
Helical model with major-minor grooves	1100	206	438	-82.0	2.40

**Fig 6:**
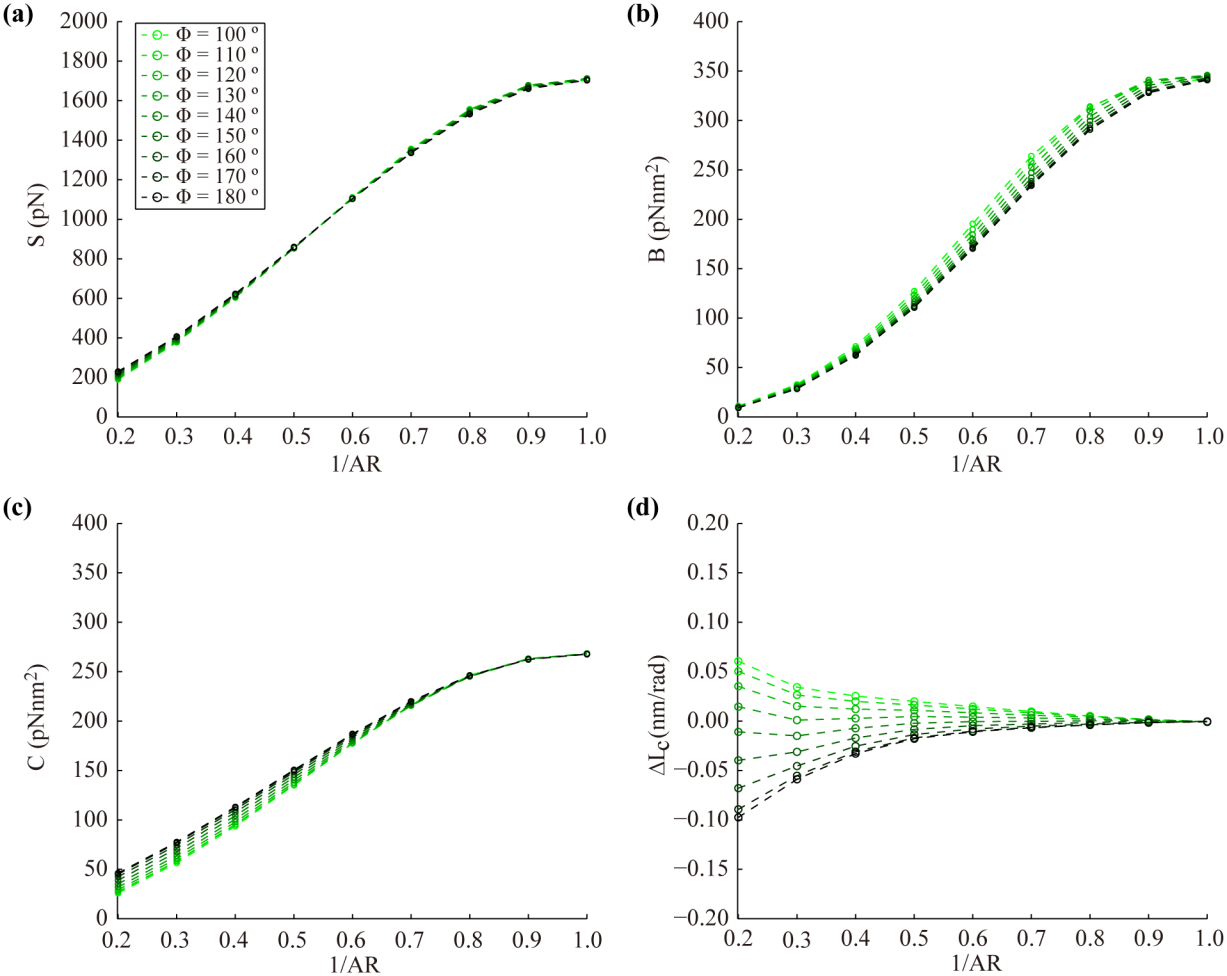
Effect of the groove angle on the mechanical properties of the bare helix model. (a-c) The groove angle has negligible effects on S, B, and C. (d) However, ΔL_c_ is sensitive to the groove angle particularly when the aspect ratio is high. Results are calculated using E_c_ = 668 MPa and D = 2.4 nm with the default helical parameters.

Hence, we first investigate the effect of major-minor grooves on the mechanical properties of the bare helix model constructed using various groove angles, Ф, ranging from 100° to 180° (Fig 6). It turns out that the duplex rigidities (S, B, and C) are hardly affected by Ф (Fig 6a–6c), which is largely because the cross-sectional area is kept constant and the size of the load-bearing core remains almost unchanged. On the other hand, Ф shows a significant influence on ΔL_c_ particularly with high AR values. As the major-minor grooves become more prominent (or Ф deviates more from 180°), ΔL_c_ increases rapidly even to positive values that are unattainable using the bare helix model without major-minor grooves (Fig 6d). Similar trends are observed when the stiff ribbons are included as well except for a reduction of C because the major-minor grooves weaken the effect of S_r_ on C (Fig E in S1 File). And yet, the major-minor grooves contribute mostly to the twist-stretch coupling of the duplex. For example, when the groove angle of the B-form DNA (Ф = 130°) is used, the increase in ΔL_c_ is observed consistently over the range of parameter values tested (Fig 5a). Therefore, the major-minor grooves play a primary structural role in controlling the twist-stretch coupling of the DNA double helix. As a result, the experimental ΔL_c_ can now be reached using a helix diameter (D = 2.2 ~ 2.4 nm) within the experimental range (Fig 5b). To illustrate, if we use the helicity and the groove angle of the B-form DNA, D = 2.4 nm, 1/AR = 0.6, E_c_ = 668 MPa and S_r_ = 1,100 pN form a feasible parameter set leading to the duplex properties of S = 1100 pN, B = 206 pNnm^2^, C = 438 pNnm^2^, and g = −82 pNnm close to the experimentally measured values [4] (Table 1).

### Effect of the helical parameters

**Fig 7:**
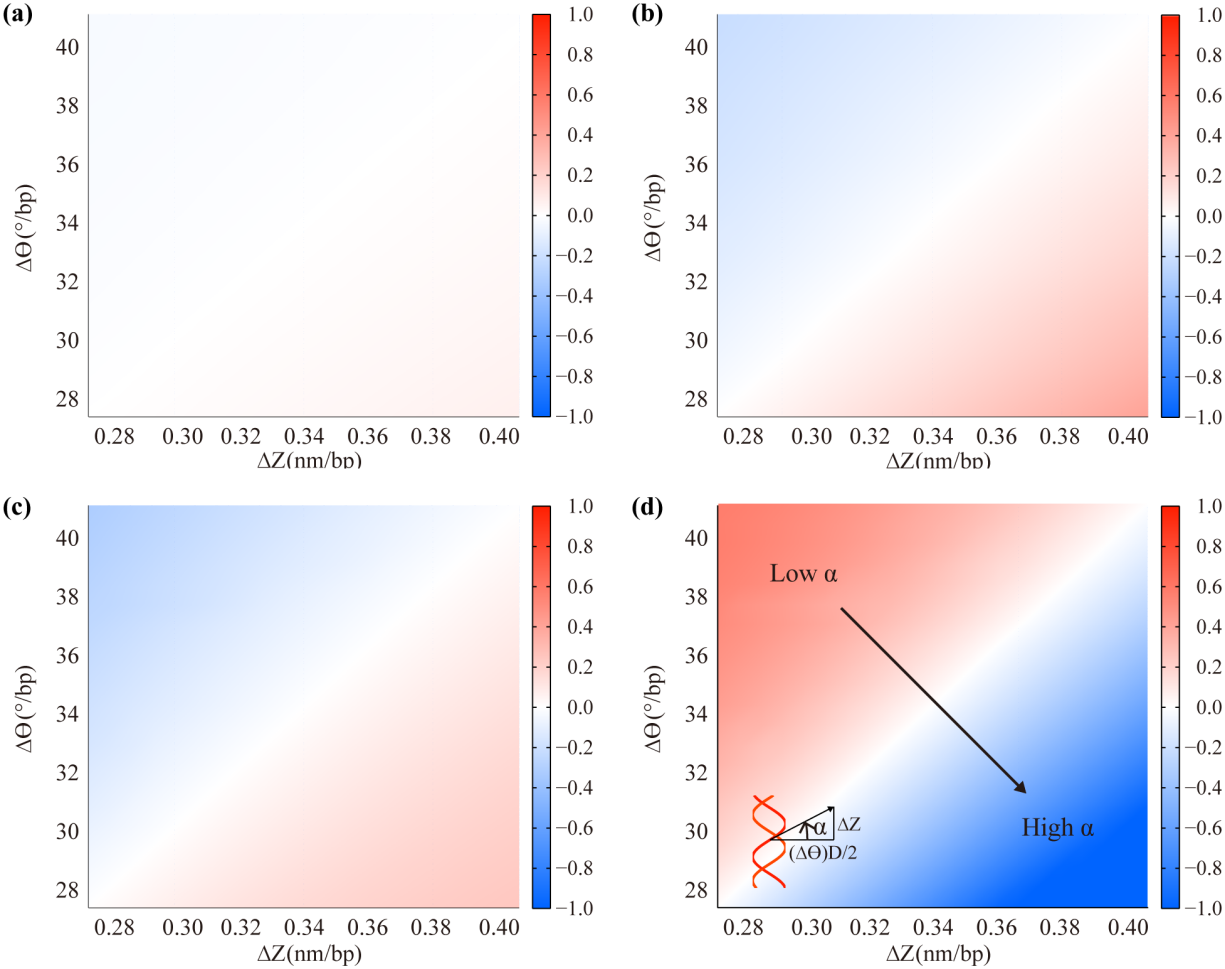
Effect of the helical parameters. Results are calculated using E_c_ = 668 MPa, D = 2.4 nm, 1/AR = 0.6, S_r_ = 1100 pN and Ф = 130°. Colors show (a) (S − S_ref_)/S_ref_, (b) (B − B_ref_)/B_ref_, (c) (C − C_ref_)/C_ref_, and (d) (ΔL_c_ − ΔL_c,ref_)/ΔL_c,ref_ where S_ref_, B_ref_, C_ref_, and ΔL_c,ref_ are the reference stretching, bending and torsional rigidities and the axial displacement coupled to twist, respectively, computed using the default helical parameters, Δθ = 34.29°/bp and ΔZ = 0.34 nm/bp. While the rigidities increase, ΔL_c_ decreases with the helix angle, α = tan^-1^(2ΔZ/DΔθ).

Finally, we look into the effect of helical geometry on the mechanical properties of the DNA double helix by varying the helical parameters, Δθ and ΔZ, up to ±20% from their default values while fixing the other parameters to the values listed above. Results demonstrate that the duplex properties are dependent on the ratio between Δθ and ΔZ corresponding to the helicity (twist angle per unit length) rather than on their individual values (Fig 7). If we define the helix angle, α = tan^-1^(2ΔZ/DΔθ), as an alternative parameter which is inversely proportional to the helicity, the duplex rigidities increase with α as expected from the effect of the helicity on these rigidities (Fig 2 and Fig C in S1 File). Both B and C change up to ±30% and ±40%, respectively, with our variation on the helicity while S shows a relatively smaller change less than ±7% (Fig 7a–7c and Fig Fa-c in S1 File). More importantly, the helix angle shows a significant influence on the twist-stretch coupling of the duplex. ΔL_c_ decreases significantly with α to the extent that even its sign can be changed while the effect of major-minor grooves on the twist-stretch coupling increases with it (Fig Fd in S1 File). This result suggests that a transition from the overtwisting state to the undertwisting state occurring under a tensile force [4, 6] might be due to the increase of the helix angle when stretched according to our model.

## Conclusions

In conclusion, we investigate the effect of principal structural features of the DNA double helix on its mechanical properties using the helical continuum model. The proposed model reproduces successfully the elastic mechanical properties of the B-form DNA measured experimentally. Our study suggests, in particular, that (1) the stiffness of phosphate backbones is essential to achieve the counterintuitive overwinding behavior of the helix under tension and contributes mostly to the extraordinarily high torsional rigidity of the duplex, (2) the major-minor grooves increase the magnitude of the twist-stretch coupling particularly at a low helicity, and (3) the twist-stretch coupling is highly sensitive to the helicity implying the possibility of its transition from the overtwisting phase to the undertwisting phase or vice versa when a sufficiently large amount of tensile force or torsional moment is applied. We anticipate that the proposed model offers a versatile tool to explore the mechanics of various helical structures in depth because, for example, it can be easily integrated with other refined modeling approaches including molecular dynamics simulations in multi-scale analysis framework, enabling us to link the local conformational changes due to external forces, base pair modifications, and binding molecules to the global structural properties.

## Supporting Information

S1 File Supporting results for finite element analysis. This file contains Figs A-F and Table A.
(PDF)
