# Supplementary material for: Structural Basis for Elastic Mechanical Properties of the DNA Double Helix
Source: PLoS One. 2016 Apr 7;11(4):e0153228. doi: 10.1371/journal.pone.0153228 (PMC4824394; doi:10.1371/journal.pone.0153228)
Supplement: S1 File — This file contains Figs A-F and Table A. (PDF) [file pone.0153228.s001.pdf]

Supporting Information

Structural Basis for Elastic Mechanical Properties of the DNA Double Helix

Young-Joo Kim[1] and Do-Nyun Kim[1,2*]

[1] Department of Mechanical and Aerospace Engineering, Seoul National University, City, Gwanak-ro 1, Gwanak-gu, Seoul, 08826, Republic of Korea.

[2] Institute of Advanced Machines and Design, Seoul National University, Gwanak-ro 1, Gwanak-gu, Seoul, 08826, Republic of Korea

* Corresponding author. E-mail: dnkim@snu.ac.kr

Fig A. Effect of the element size

Fig B. Effect of the helix length on the calculated mechanical properties

Fig C. Effect of the helicity on the duplex rigidities in the bare helix model

Fig D. Effect of the backbone stiffness on the mechanical properties of the helical model at several D values

Fig E. Effect of the groove angle on the mechanical properties of the helical model with backbone stiffness

Fig F. Effect of α on the mechanical properties

Table A. Summary of the parameters used in helical continuum models

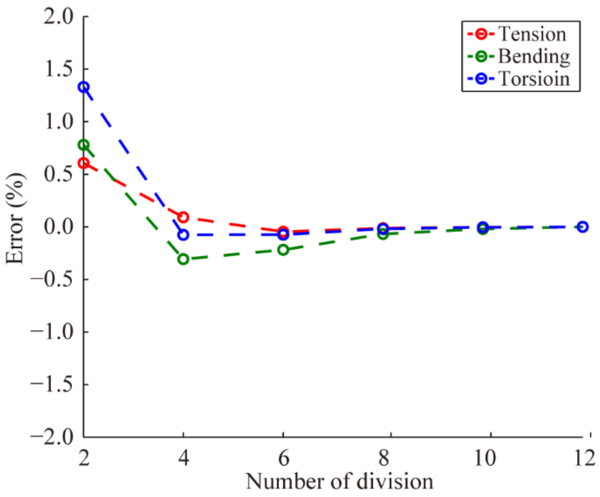

Fig A. Effect of the element size. The horizontal axis represents the number of division per axial rise along the helical axis (n) controlling the element size while the vertical axis shows the strain energy error. Errors in tensile (red), bending (green) and torsional (blue) loadings are calculated for various n using the strain energy for n = 12 as a reference. For computational efficiency, n = 6 is used in our analysis resulting in errors less than 0.3%. These results are obtained for the bare helix model without backbone stiffness using E_c = 668 MPa, D = 2.4 nm and 1/AR = 0.6 with the default helical parameters.

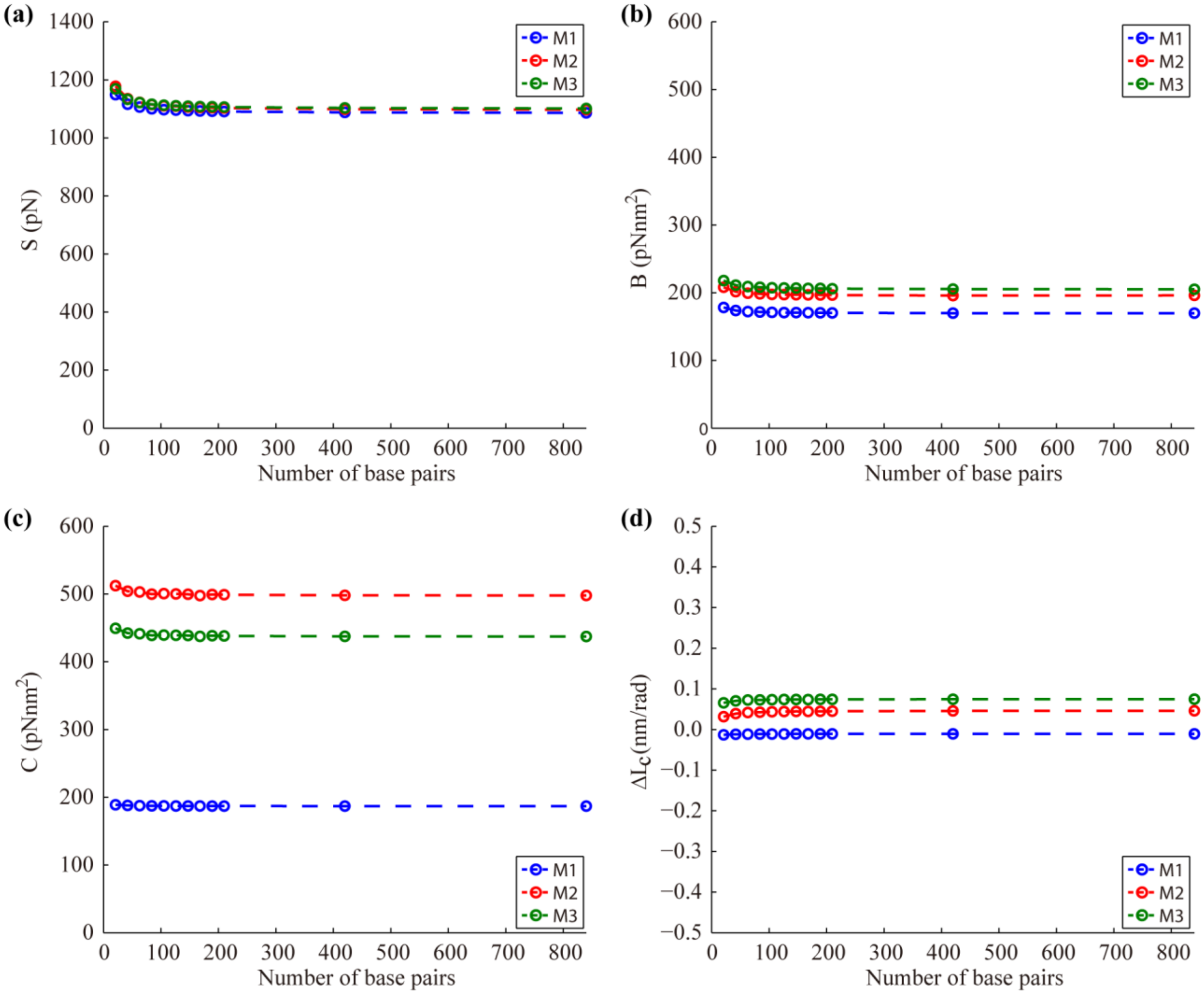

Fig B. Effect of the helix length on the calculated mechanical properties. The mechanical properties for the helical models whose length varies from 21 to 840 base pairs are computed using E_c = 668 MPa, D = 2.4 nm, 1/AR = 0.6, and S_r = 1100 pN with the default helical parameters. M1 (blue), M2 (red) and M3 (green) represent the results for the bare helix model, the helical model without major-minor grooves and the helical model with major-minor grooves, respectively. Results demonstrate that the length does not affect the solutions as long as the helix is longer than 100 base pairs.

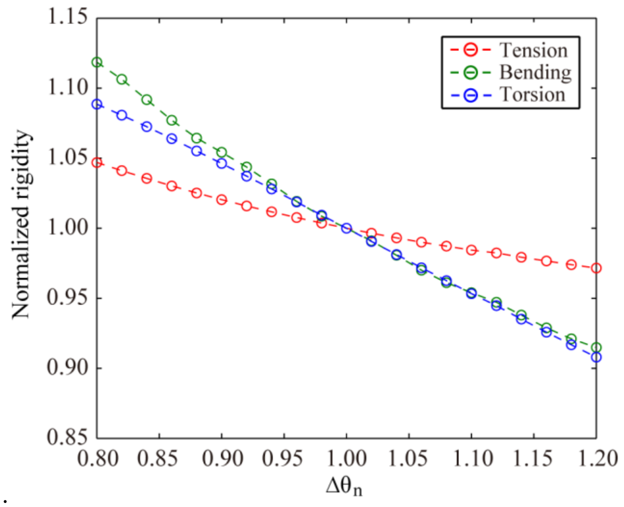

Fig C. Effect of the helicity on the duplex rigidities in the bare helix model. Rigidities are computed using E_c = 668 MPa, D = 2.4 nm, and 1/AR = 0.6 with the default axial rise. $\Delta\theta_n$ represents $\Delta\theta/\Delta\theta_{ref}$ where $\Delta\theta$ is the twist rate and $\Delta\theta_{ref}$ is its default value. Rigidities are normalized by those calculated using $\Delta\theta_{ref}$.

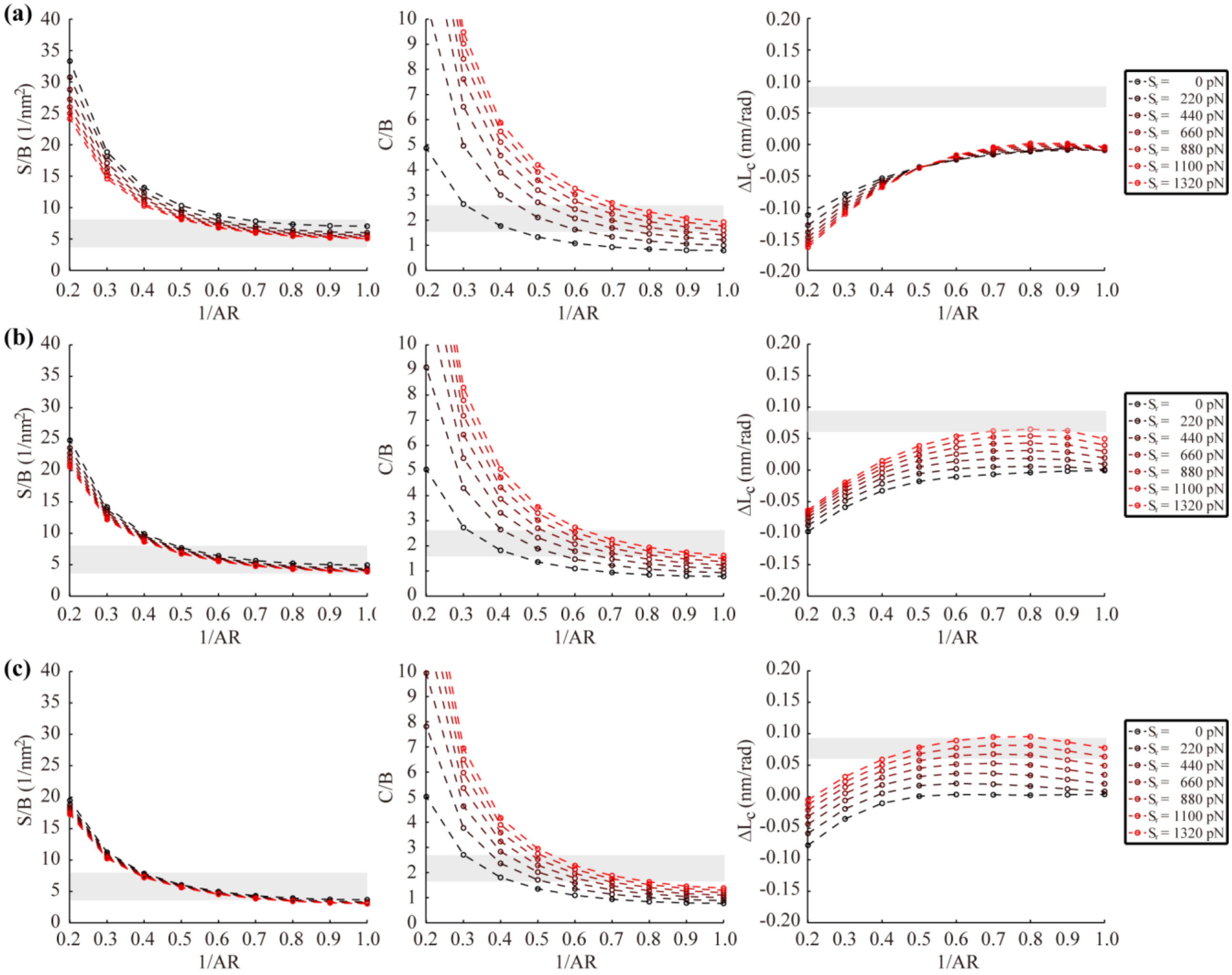

Fig D. Effect of the backbone stiffness on the mechanical properties of the helical model at several D values (a) D = 2.0 nm, (b) D = 2.4 nm and (c) D = 2.8 nm. Results are calculated using the default helical parameters. Shaded regions represent the range of experimental values. Decrease of S/B due to stiff ribbons becomes smaller as D increases. It means that increase of B becomes significant due to stiff ribbons when bending rigidity of a helical core is relatively small compared to that of stiff ribbons in their width direction.

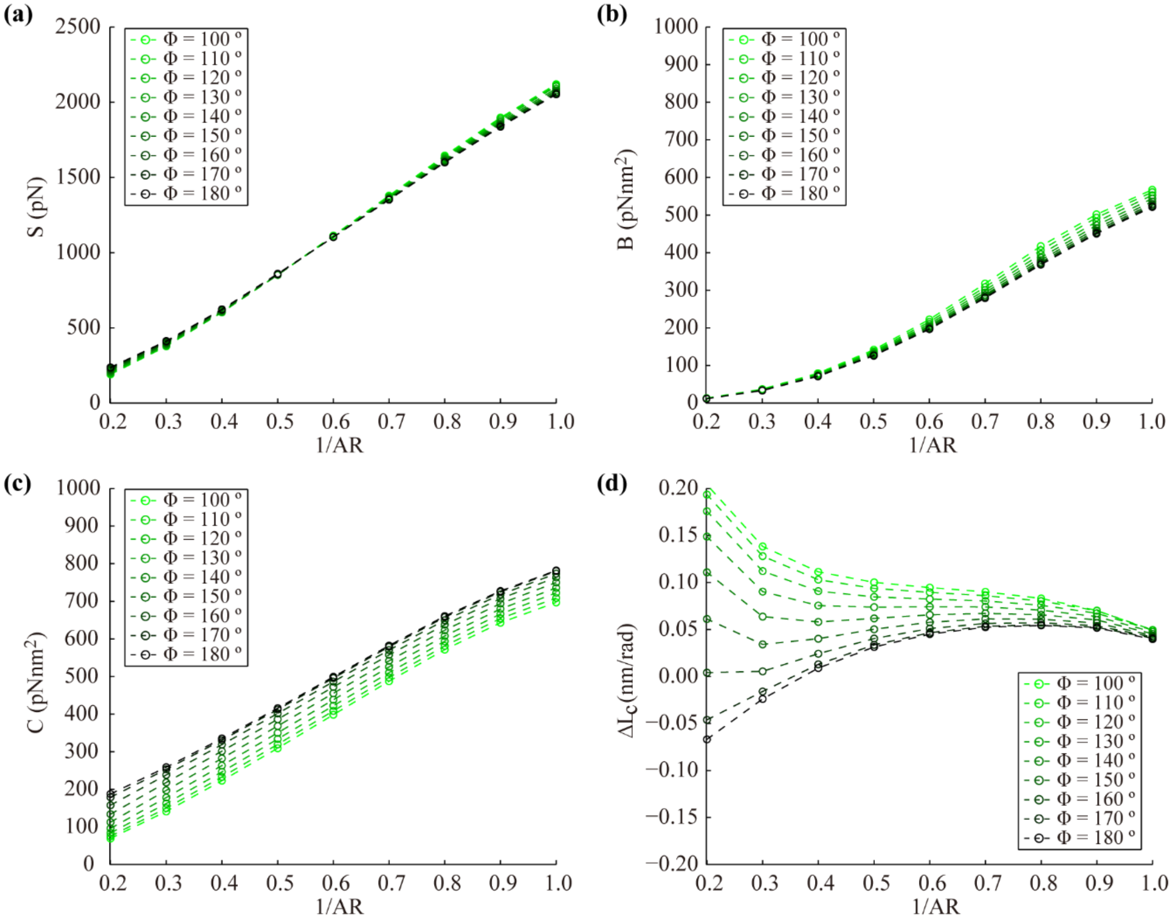

Fig E. Effect of the groove angle on the mechanical properties of the helical model with backbone stiffness. Results are calculated using E_c = 668 MPa, D = 2.4 nm, and S_r = 1100 pN with the default helical parameters.

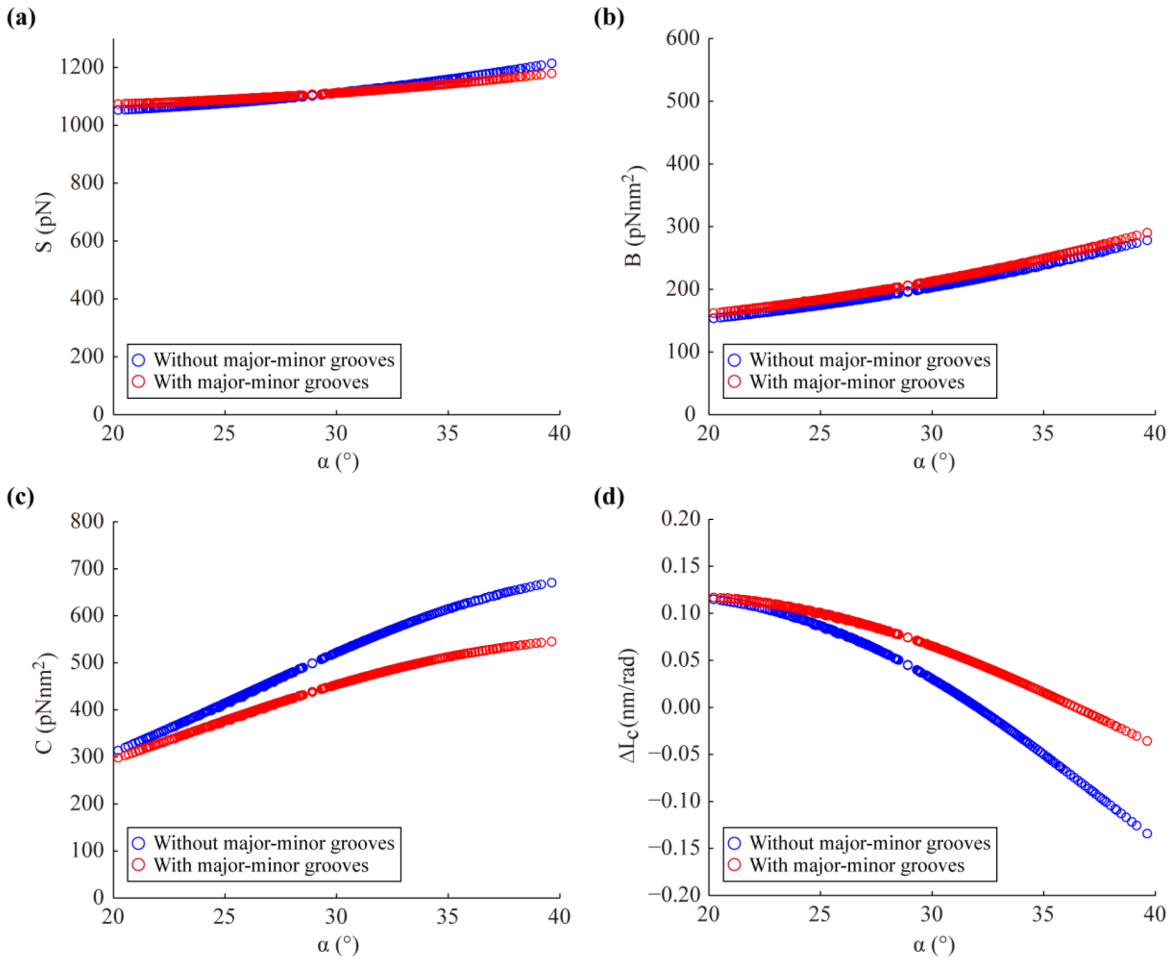

Fig F. Effect of α on the mechanical properties. Results are calculated using E_c = 668 MPa, D = 2. 4 nm, 1/AR = 0.6, and S_r = 1100 pN.

Table A. Summary of the parameters used in helical continuum models

Parameters					
Cross-section	D	W	H	AR	Φ
	Diameter	Width	Height	Aspect ratio (=W/H)	Groove angle
Helical shape	ΔZ		Δθ		α
	Axial rise		Twist rate		Helix angle (=$\tan^{-1}(2\Delta Z/\Delta\theta)$)
Material	E_c		ν_c		S_r
	Young's modulus of the core helix		Poisson's ratio of the core helix		Stretching rigidity of the stiff ribbons
